# Water‐Soluble Squaramide‐Functionalized Copolymers for Anion Recognition

**DOI:** 10.1002/marc.202300406

**Published:** 2023-10-02

**Authors:** Jakob D. E. Lane, Gabrielle Shiels, Parathan Ramamurthi, Markus Müllner, Katrina A. Jolliffe

**Affiliations:** ^1^ School of Chemistry The University of Sydney NSW 2006 Australia; ^2^ Key Centre for Polymers and Colloids School of Chemistry The University of Sydney NSW 2006 Australia; ^3^ The University of Sydney Nano Institute (Sydney Nano) The University of Sydney NSW 2006 Australia; ^4^ Australian Research Council Centre of Excellence for Innovations in Peptide and Protein Science The University of Sydney NSW 2006 Australia

**Keywords:** ATRP, fluorescence sensors, H‐bonding, PEG, post‐functionalization

## Abstract

A series of ethylene glycol‐based squaramide‐containing copolymers are synthesized via a post‐polymerization functionalization strategy. Conversion of polymeric amines to squaramides is found to proceed in good yields, representing a versatile method of incorporating squaramides into polymers for anion recognition. Analysis of the polymers by UV‐Vis and fluorescence spectroscopy revealed that anion binding takes place similarly to that of small‐molecule squaramides. The presence of a fluorescent sensing group on polymer‐bound squaramides allowed for a fluorescent sensing mechanism for anions that followed a similar trend in selectivity in aqueous DMSO solution, with selectivity observed for H_2_PO_4_^−^, AcO^−^ and SO_4_^2−^ over other common anions tested. The anion response and selectivity towards anions is similar to that of analogous small‐molecule squaramides, however polymeric squaramides exhibited a greater resistance to deprotonation by more basic anions, which is attributed to the closer proximity of individual squaramides on a macromolecule. The squaramide‐containing polymers exhibited good water solubility, overcoming a common problem for anion sensors which are typically not sufficiently soluble in water to function in many required applications. Despite no anion binding being observed in water, this study represents a simple and effective method of creating fully water‐soluble anion receptors which may be adapted to give improved binding affinity and selectivity depending on the anion binding moiety.

## Graphical abstract

A series of ethylene glycol‐based squaramide‐containing copolymers are synthesized via a post‐polymerization functionalization strategy, representing a new method of incorporating squaramides into polymers for anion recognition and sensing.

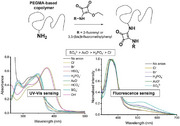

## 1 Introduction

Selective anion recognition and sensing has generated significant interest in recent years due to the high abundance of anionic species in biological and industrial processes, in addition to anions comprising notable industrial and agricultural pollutants.^[^ ^1^, ^2^, ^3^, ^4^ ^]^ Thus, the development of new binding motifs and frameworks continues to be an active area of research, in order to achieve systems with increased binding affinity, selectivity towards anions, and improved physical properties of the receptors such as (water) solubility.^[^ ^5^, ^6^, ^7^, ^8^, ^9^, ^10^, ^11^, ^12^ ^]^

Hydrogen bonding has proven to be an effective means of interaction for synthetic anion receptors, with high binding affinities and good selectivity achieved in water‐containing media.^[^ ^6^, ^13^, ^14^, ^15^, ^16^, ^17^, ^18^ ^]^ Commonly used H‐bonding motifs include the dual H‐bonding (thio)ureas and squaramides as they exhibit two directional H‐bonds, and have been shown to give inherent selectivity towards anions that provide a good geometric match, such as carboxylates and sulfate. There are many examples of these motifs being incorporated into more complex receptor structures such as peptides or macrocycles, for applications such as anion sensing or anion extraction, which demonstrates their versatility.^[^ ^2^, ^19^, ^20^ ^]^

There have been multiple reported examples of polymers containing dual H‐bonding motifs such as ureas and thioureas for anion recognition.^[^ ^21^, ^22^, ^23^, ^24^ ^]^ Rarer, however, are examples of polymeric materials containing the squaramide motif for anion recognition.^[^ ^25^, ^26^, ^27^ ^]^ Squaramides have been demonstrated to give rise to improved anion affinity and improved anion transport efficacy compared with (thio)ureas, and thus represent an important tool for anion recognition.^[^ ^28^ ^]^ For most anion recognition applications, it is useful for the receptor to function in water, which has often proven challenging due to the inherent low solubility of small molecule anion receptors, and the competitive H‐bonding nature of water. Appending water solubilizing groups, such as poly(ethylene glycol) chains to anion recognition motifs typically increases synthetic complexity and can make purification difficult.^[^ ^6^ ^]^ We envisaged that an inherently water‐soluble polymer framework could help to overcome these synthetic challenges and allow relatively hydrophobic motifs to be readily solubilized in water. Furthermore, the unique physical properties of polymers compared to small molecules (such as the ability to respond to external stimuli including temperature, humidity, pH, electrical, or irradiation) may be exploited for novel anion sensing or extraction mechanisms in the future.^[^ ^29^, ^30^, ^31^, ^32^, ^33^, ^34^, ^35^, ^36^ ^]^ Therefore, we sought to develop a post‐polymerization method to append squaramides to linear copolymers and to investigate their anion binding capabilities.

Previous examples of squaramide‐containing polymers for anion recognition have relied upon the polymerization of squaramide‐containing monomers, either via step‐growth or chain‐growth polymerization techniques.^[^ ^25^, ^27^ ^]^ These strategies necessitate the synthesis of squaramide‐containing (co)monomers, which can add synthetic complexity and may limit the scope of the squaramides or anion receptors that can be incorporated into a polymer framework.

In this study, a new strategy for the synthesis of anion‐binding polymers was investigated whereby copolymers were functionalized with squaramides post‐polymerization. Their water solubility was confirmed and their ability to bind to and sense anions was subsequently demonstrated in aqueous DMSO solution. Copolymers containing varying ratios of poly(ethylene glycol) methyl ether methacrylate (PEGMA, *M*_n_  = 300 g mol^−1^) and [(*tert*‐butoxycarbonyl)amino] ethyl methacrylamide (Boc‐AEMA) were synthesized via atom transfer radical polymerization (ATRP). PEGMA‐based polymers are highly hydrated, ensure a good water solubility of copolymers, and can be easily copolymerized with functional handles.^[^ ^37^, ^38^, ^39^ ^]^ The *tert*‐butoxycarbonyl protecting groups were subsequently removed to yield free amines, which were functionalized by a standard squaramide synthesis to obtain polymers containing pendant squaramide motifs. The anion binding properties of the squaramide‐appended polymers were investigated via UV‐Vis anion screens and titrations to determine the similarity of anion binding compared to the analogous small‐molecule squaramides, and the selectivity of binding towards different anions. Finally, the anion sensing capability of a fluorescent fluorenyl squaramide‐containing copolymer was investigated via fluorescence anion screens, to assess anion response and selectivity.

## 2 Results and Discussion

### 2.1 Synthesis of Squaramide‐Functionalized Copolymers

In order to synthesize copolymers containing squaramide motifs, firstly squaramates **Sqt1** and **Sqt2**, containing 3,5‐(bis)trifluoromethylphenyl and fluorenyl substituents, were synthesized from diethyl squarate and the corresponding amines following adapted literature procedures (Scheme S1, Supporting Information).^[^ ^28^, ^40^ ^]^ These substituents were chosen to demonstrate that squaramide derivatives of varying functionality may be incorporated into polymers post‐polymerization. The 3,5‐(bis)trifluoromethylphenyl substituent is highly electron withdrawing and as a result provided relatively acidic squaramide NH protons to enhance anion binding affinity, while the 2‐fluorenyl substituents gave rise to acidic squaramide NH protons while also acting as a fluorophore to allow detection of anion binding by fluorescence spectroscopy.^[^ ^41^, ^42^ ^]^ Squaramides **Sq1** and **Sq2** (Scheme S2, Supporting Information) were also synthesized to provide an analogous small molecule comparison to the polymeric squaramides in anion binding investigations. As it was desirable to obtain copolymers of a controllable molecular weight and low dispersity to better compare between small molecule and polymeric squaramides, we used ATRP to first synthesize our copolymers and subsequently functionalized them with squaramides post‐polymerization. Reversible deactivation radical polymerization methods, like ATRP, have continuously shown to excel in the synthesis of functional macromolecular precursors for various applications.^[^ ^37^, ^43^ ^]^ Our synthetic strategy allowed for different squaramide‐containing polymers to be synthesized rapidly from the same copolymer precursor, and a diverse range of functionalized copolymers to be obtained that possess similar monomer ratio, dispersity, and molecular weight.

We identified Boc‐AEMA (containing a protected amine) as a useful comonomer to provide an amine handle for post‐polymerization modification (Scheme S3, Supporting Information).^[^ ^44^, ^45^, ^46^ ^]^ Boc‐AEMA was then copolymerized with PEGMA in a 1:5 monomer ratio to produce polymer **P1** (Scheme S4, Supporting Information). Polymer **P2** was similarly prepared through co‐polymerization of Boc‐AEMA and PEGMA in a 1:10 monomer ratio. The ratio of monomers in the polymers obtained was found to be similar to the stoichiometry in the feedstock by analysis of the relative integrals of the characteristic ^1^H NMR signals (the signal attributed to the *tert*‐butyloxycarbonyl group for the Boc‐AEMA monomer, 1.41 ppm, and the methoxy group for the PEGMA monomer, 3.38 ppm, Section 5, Supporting Information).

**Scheme 1:**
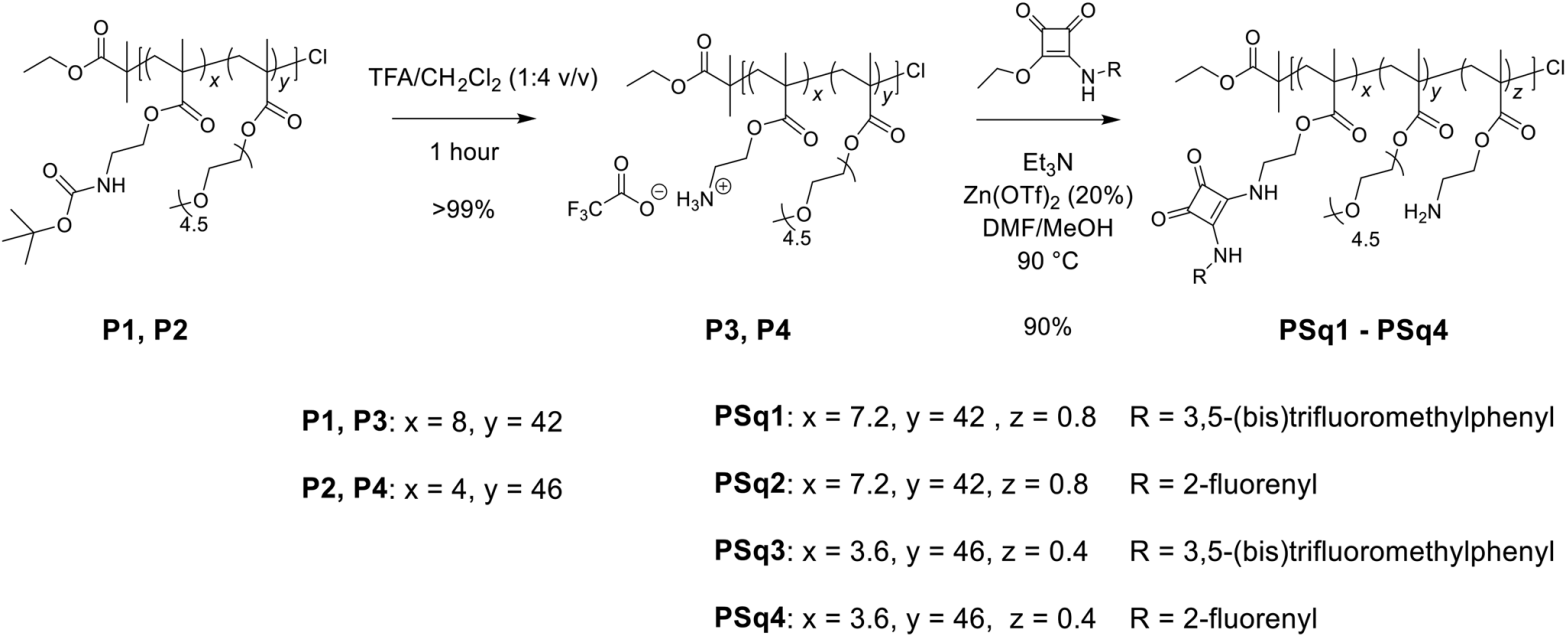
Synthesis of squaramide‐containing polymers via a post‐polymerization functionalization strategy of PEGMA‐based copolymer precursors.

To functionalize the polymers, the *tert*‐butyloxycarbonyl protecting groups were removed upon treatment with trifluoroacetic acid (TFA) in CH_2_Cl_2_ (1:4 *v/v*) (**Scheme** **1**). Complete deprotection of the amines was confirmed by the absence of the signal attributed to the *tert*‐butyl CH_3_ protons (1.41 ppm) in the ^1^H NMR spectra of the resultant polymers. Squaramide functionalization of the polymers was achieved using an adapted method for squaramide synthesis through reaction with either **Sqt1** or **Sqt2** in a mixture of DMF:MeOH (1:1).^[^ ^40^ ^]^ Both the TFA salts and the free amines could be successfully functionalized in this way, providing Et_3_N was added to the reaction to ensure the amines were not protonated. Optimization of the reaction conditions was undertaken to achieve high conversion of amine to squaramide (Table S1, Supporting Information), as it was anticipated that remaining free amines may interfere with anion binding or react with the polymer and cause degradation. Ultimately, the yield obtained for functionalization of polymers with squaramides was comparable or superior to that of solution phase squaramide synthesis.^[^ ^40^ ^]^ This may be attributed to ease of purification for polymeric squaramide synthesis, which was done via dialysis in acetone/DMF (1:1, *v/v*), and the excess of squaramate that was used in order to obtain maximum conversion.

The size exclusion chromatography (SEC) traces for all polymers synthesized in this study are shown in **Table** **1**. ^1^H NMR analysis of each polymer was used to determine their molecular weights. The complete consumption of monomers from the polymerization of polymers **P1** and **P2** indicated that the degree of polymerization was 50 in each case. As described above, a comparison of the signal attributed to the *tert*‐butyloxycarbonyl group of the Boc‐AEMA, and the methoxy group for the PEGMA allowed the ratio of Boc‐AEMA and PEGMA monomers to be determined. Subsequently, for squaramide appended polymers **PSq1** – **PSq4**, the degree of functionalization was calculated by comparison of the integration of aromatic signals of the squaramide substituents with the known monomeric ratio of the AEMA and PEGMA.

**Table 1:** Overview of the synthesized squaramide‐containing copolymer library

Copolymer	Composition^a)^ (PEGMA: BocAEMA)	*M*_n, NMR_ ^a)^ (Da)	*M*_n, SEC_ ^b)^ (Da)	Ð^b)^
P1	5:1	14 300	10 400	1.09
P2	10:1	14 700	9 800	1.10
P3 (P1 deprotected)	5:1	13 300	11 100	1.11
P4 (P2 deprotected)	10:1	14 400	8 900	1.11
PSq1	5:1	16 400	22 500	1.17
PSq2	10:1	15 500	10 800	1.14
PSq3	5:1	15 900	21 000	1.13
PSq4	10:1	15 400	12 100	1.08

^a)^ obtained from ^1^H NMR, >99% monomer conversion

^b)^ obtained from SEC in DMAc (50°C and 1 mL min^–1^) using PMMA standards.

**Figure 1:**
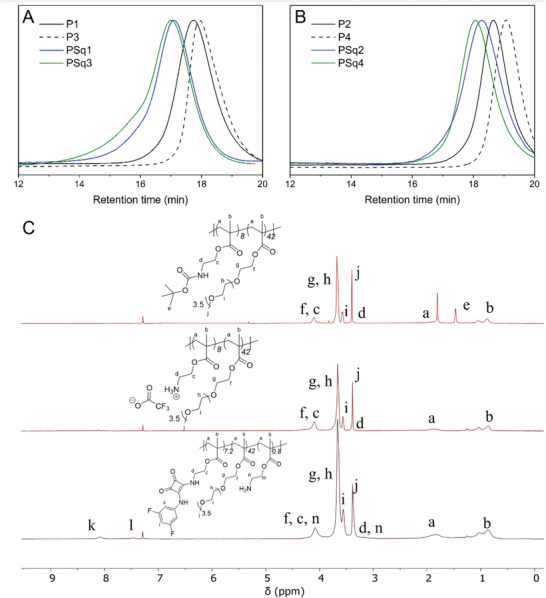
A,B) SEC elugrams for copolymers synthesized in this study. C) Representative ^1^H NMR spectra (CDCl_3_, 400 MHz) of **P1**, **P3** and **PSq1** showing changes upon Boc deprotection (loss of signal at 1.46 ppm), and squaramide functionalization (aromatic signals at 8.11 and 4.66 ppm).

Polymers of different Boc‐AEMA:PEGMA ratios, with either 1:5 (**PSq1** and **PSq3**), or 1:10 (**PSq2** and **PSq4**), were synthesized to allow an investigation into whether a greater number of squaramides per polymer would lead to any differences in binding or selectivity. Our method of polymer functionalization proved useful for the installation of different squaramides, which were synthesized in comparable conversion to the yields observed for the small molecule squaramide analogues: **PSq1** and **PSq3** featuring 3,5‐(bis)trifluoromethylphenyl squaramides and **PSq2** and **PSq4** featuring 2‐fluorenyl squaramides. Our methodology demonstrates how post‐functionalization can be used to prepare a modular library of functionalized polymers with varied anion recognition moieties. The purity of the modified polymers and the conversion of squaramide formation were determined by ^1^H NMR and SEC (Table 1, **Figure** **1**, and Section S5, Supporting Information). All polymers were found to be soluble in water at room temperature, representing a promising platform for the development of squaramide‐containing anion receptors.

### 2.2 Anion Binding Studies

**Figure 2:**
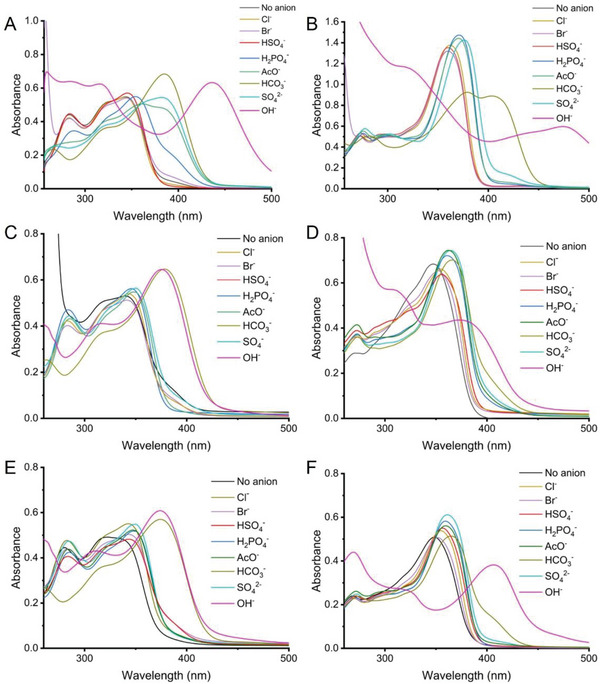
UV‐Vis anion screens of A) **Sq1** and B) **Sq2** in DMSO (1% water), with squaramide concentration of 25 µM and anion concentration of 1.25 mM. UV‐Vis anion screens of copolymers C) **PSq1**, D) **PSq2**, E) **PSq3**, F) **PSq4** in DMSO (1% water), with squaramide unit concentration 18 – 25 µM and anion concentration 0.90 – 1.25 mM. All anions added TBA^+^ salts except HCO_3_^−^ which was added as TEA^+^.

Having obtained our squaramide containing copolymers **PSq1** – **PSq4**, we sought to assess their anion binding properties for a range of anions by conducting anion screens in aqueous DMSO solution (**Figure** **2**). The change in electronics of the aromatic substituents (either 3,5‐(bis)trifluoromethyl or fluorenyl) upon H‐binding to anions can be monitored via UV‐Vis spectroscopy. A bathochromic shift and change in absorption intensity are typically observed in the UV‐Vis spectrum upon anion binding.^[^ ^7^, ^42^ ^]^ Therefore, anion screens at 50 equivalents of anion in DMSO (1% water) were carried out to determine which anions the polymers bind more strongly to. Anions were added as their tetrabutylammonium (TBA^+^) salt as this is a large, non‐coordinating cation that has minimal effects on the receptor – anion binding (HCO_3_^−^ was added as the tetraethylammonium, TEA^+^, salt). To obtain the best comparison, the concentration of squaramide was kept constant with the squaramide concentration for the monomeric analogs. For most anions, the changes in the UV‐Vis spectra of the polymer‐bound squaramides were largely consistent with those observed for the small‐molecule squaramide analog, further confirming that anion binding was taking place in the polymeric materials.

Small‐molecule squaramides **Sq1** and **Sq2** were first screened against a range of anions under the same conditions to provide a comparison to the copolymer squaramides (Figure 2). These screens indicated that most monovalent anions caused minimal changes to the absorbance spectra, indicative of weak anion binding. By contrast, for all copolymers, the shift in absorbance was largely consistent with the small molecule squaramides, and indicated that SO_4_^2−^, AcO^−^, H_2_PO_4_^−^ and HCO_3_^−^ were bound most strongly. For small‐molecule receptors **Sq1** and **Sq2**, the addition of OH^−^ resulted in deprotonation of the NH protons, evidenced by the significant shift in of λ_max_ to 450 nm for **Sq1** (Figure 2a) and 485 nm for **Sq2** (Figure 2b), which was notably different to the shifts observed for anion binding.^[^ ^40^, ^47^ ^]^ In contrast, the addition of OH^−^ did not result in the same significant shift of λ_max_ for the 3,5(bis)‐trifluoromethylphenyl squaramide‐functionalized copolymers, **PSq1** and **PSq3**, suggesting that deprotonation was less facile for the polymeric squaramides. This may be attributed to the relative proximity of squaramides on the polymer chain making complete deprotonation of all squaramides less likely due to the p*K*_a_ of the acidic protons being influenced by their surroundings, causing the overall polymer to behave like a polyelectrolyte.^[^ ^48^, ^49^ ^]^

A similar difference in the response to OH^−^ for fluorenyl squaramide‐containing copolymers in comparison to small‐molecule **Sq2** was observed, with the polymeric squaramides showing an apparent resistance to complete deprotonation. The fluorenyl squaramide‐containing copolymers, **PSq2** and **PSq4**, showed a difference in the response to HCO_3_^−^ compared to the small‐molecule analogue (Figure 2b,d,e). **Sq2** appeared to undergo deprotonation in the presence of HCO_3_^−^, with the absorbance maximum shifting to 420 nm. In the analogous polymeric systems, the response to HCO_3_^−^ was similar to that of other oxoanions (such as SO_4_^−^ and AcO^−^), indicating that binding was likely taking place between the polymeric squaramide and HCO_3_^−^ rather than deprotonation. For copolymers of different monomeric ratios, the response to anions was similar. Anion titrations were carried out for squaramides **Sq1** and **Sq2** (Figures S1,S2, Supporting Information) and the data fit to a 1:1 binding model, the binding affinity trend is Cl^−^ > H_2_PO_4_^−^ > AcO^−^ > SO_4_^2−^, and the magnitude of changes in the UV‐Vis spectra during these screens indicated that this trend was true for copolymers **PSq1** – **PSq4**.^[^ ^50^, ^51^, ^52^ ^]^ Titrations performed under the same condition with the polymeric **PSq1** and the same four anions showed similar trends to those observed for the monomeric analog, with addition of acetate and sulfate giving the largest changes. (Figure S3, Supporting Information). The addition of anions caused a red shift in the absorbance maxima, and a decrease in absorbance at 360 nm with increased absorbance at longer wavelength. While the data could not be fitted to a binding model, the magnitude of the changes indicates the trend in binding affinities mirrors that of **Sq1**.^[^ ^50^, ^51^ ^]^ Despite the squaramide‐containing copolymers being fully soluble in water, no response was observed by UV‐Vis upon addition of anions as either their TBA^+^ or Na^+^ salts in pure water, indicating that these copolymer squaramides were still unable to interact strongly with anions in a highly competitive H‐bonding solvent.

The presence of the fluorene substituent on squaramide **Sq2** and polymeric squaramides **PSq2** and **PSq4** allowed for the anion‐sensing properties of the copolymers to be investigated via fluorescence spectroscopy. Fluorescence is a preferred method of sensing in a range of fields due to its sensitivity at low concentrations of analytes and the practicality of use.^[^ ^13^, ^53^, ^54^ ^]^ Fluorophore containing‐squaramides have been shown to be useful for obtaining a fluorescent response to anions, and therefore as potential anion‐sensing moieties. Recently, squaramides conjugated to the fluorene group have been reported to give a fluorescent response to anions, with selectivity observed towards acetate, dihydrogen phosphate, and sulfate.^[^ ^42^ ^]^

**Figure 3:**
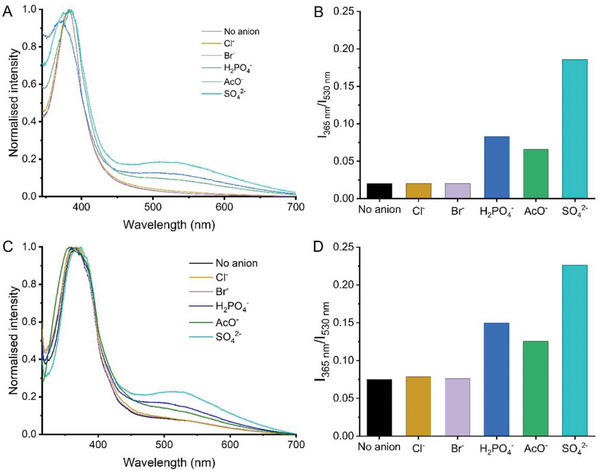
A) Fluorescence screen of **Sq2** (20 µM) with anions (1 mM, 50 equivalents) in DMSO (1% water), excitation 309 nm. B) Fluorescence emission ratio I_365 nm_/I_530 nm_, **Sq2** (20 µM) in the presence of different anions (1 mM, 50 equivalents) in DMSO (1% water). C) Fluorescence screen of **PSq2** (20 µM of squaramide unit) with anions (1 mM, 50 equivalents) in DMSO (1% water), excitation 309 nm. D) Fluorescence emission ratio I_365 nm_/I_530 nm_, **PSq2** (20 µM of squaramide unit) in the presence of different anions (1 mM, 50 equivalents) in DMSO (1% water).

In our study, anions were screened at 50 equivalents as their TBA^+^ salts against **Sq2** and **PSq2,** and the fluorescence emission response measured. With an excitation wavelength of 309 nm, an emission band was observed at 365 nm for both small‐molecule and polymeric squaramides. Upon the addition of certain anions, an additional emission at 530 nm developed to the original emission band observed at 365 nm. The broad emission at longer wavelength was likely due to a twisted intramolecular charge‐transfer (TICT) mechanism between the anion bound squaramide and the fluorene, which has been suggested to account for these observations in fluorene containing‐squaramides.^[^ ^42^, ^55^ ^]^ The spectra were normalized to the emission at 365 nm to assess their selective response to anions (**Figure** **3**). When normalized to the emission at 365 nm, the emission spectra indicated a relative increase in the emission intensity at 530 nm upon addition of oxoanions (AcO^−^, H_2_PO_4_^−^, SO_4_^2−^), with the greatest relative turn‐on observed for SO_4_^2−^. Both small molecule and polymeric squaramides showed similar behavior and selectivity in this study, which is consistent with the changes observed in the UV‐Vis spectra upon addition of these anions. The type of fluorescence response observed for **PSq2** upon addition of oxoanions was similar to that of the small‐molecule squaramide **Sq2**, indicating a similar mechanism of fluorescence emission is taking place in both the small molecule and polymeric squaramides.^[^ ^42^ ^]^ The addition of Cl^−^ caused a minimal relative increase in the emission at 530 nm. This response to Cl^−^ is similar to the small changes observed in the UV‐Vis screens, indicating that only weak interactions were taking place between the squaramide and anions.

In relatively competitive organic solvents, a selective fluorescence response was observed for oxoanions, with some selectivity towards more basic anions (AcO^−^, H_2_PO_4_^−^) and those of higher charge (SO_4_^2−^). Given that an increase in fluorescence intensity was observed at 530 nm for several of the anions, the emission ratio *I*_365 nm_/*I*_530 nm_ was determined and identified as a means of highlighting the selectivity of this system for anion recognition. SO_4_^2−^ caused the greatest relative increase in emission at 530 nm (Figure 3), indicating a selectivity in response for SO_4_^2−^ over other anions. This also mirrored the trend in binding affinity of the small‐molecule squaramide **Sq2**. The fluorene‐containing squaramide, **Sq3**, showed very poor water solubility, hindering its potential use for anion sensing in aqueous samples. However, the fluorene squaramide appended copolymers (**PSq2** and **PSq4**) were fully water soluble, thus offering a potential pathway for the development of squaramide‐based anion sensors for use in aqueous media. However, consistent with the UV‐vis studies, no fluorescence response was observed upon addition of anions in pure water. This can again be attributed to the highly competitive nature of water as a solvent where the H‐bonding of water to the anions effectively competes with the squaramide H‐bonding.

## 3 Conclusions

We developed a simple methodology to modify copolymers with defined molecular weight and dispersity with anion‐binding squaramide motifs with high conversion. The squaramide‐containing polymers were found to be soluble in water which overcame a significant challenge in the synthesis of water‐soluble squaramide‐containing anion receptors. The response of the polymeric squaramides to anions was measured via UV‐Vis and fluorescence titrations and screens, which in turn confirmed anion binding in aqueous DMSO, with observed selectivity towards dihydrogen phosphate, acetate, and sulfate. While no anion binding was observed in pure water, our strategy for the synthesis of polymeric anion receptors may be readily adapted in the future to append a wider range of squaramides or other anion recognition moieties to inherently water‐soluble copolymers. We envisage that appending anion recognition motifs with greater pre‐organization, such as squaramide‐containing macrocycles,^[^ ^6^, ^56^ ^]^ capable of delivering a greater number of H‐bonds to the anion, may allow for designing advanced polymer‐based anion receptors that can function in purely aqueous systems, allowing a more straightforward development of potent materials for anion recognition and sensing that may find use in environmental, biological, or industrial applications.

## 4 Experimental Section

### Synthesis of Copolymers Poly{[Poly(Ethylene Glycol) Methyl Ether Methacrylate]‐co‐(2‐BOC‐Amino)Ethyl Methacrylate} (**P1**, **P2**)

For **P1**, Boc‐AEMA (180 mg, 0.785 mmol), PEGMA (900 mg, 3 mmol), E‐Bibb (11.1 uL, 0.08 mmol) and PMDETA (21.1 uL, 0.08 mmol) were dissolved in anisole (5 mL). For **P2**, Boc‐AEMA (25 mg, 0.11 mmol), PEGMA (327 mg, 1.09 mmol), E‐Bibb (3.5 uL, 0.02 mmol) and PMDETA (6.7 uL, 0.02 mmol) were dissolved in anisole (5 mL). Each reaction solution was then transferred to a Schlenk flask and degassed via three freeze‐pump‐thaw cycles. On the third freeze cycle, copper(I) chloride (10.3 mg, 0.08 mmol for P1 or 2.4 mg, 0.02 mmol for P2) was added to the flask whilst under a gentle nitrogen gas flow, and the cycle was then allowed to continue. On the third thaw cycle, the flask was backfilled with nitrogen to ensure an inert atmosphere and then placed in a heated oil bath to react at 65 °C for 24 h. After the allotted time, the reaction was stopped by cooling the flask and opening it to air. Copper was removed by passing the mixture through a silica gel. The polymer was extracted by two cycles of precipitation and re‐dissolution in cold hexane and anisole respectively. Monomer conversion: P1 >99%, P2 >99%.

### Boc Deprotection of Copolymers (**P3**, **P4**)

Boc‐protected copolymers (200 mg) were stirred in a solution of TFA/DCM (1:4, *v/v*) at 25 °C. After one hour, the TFA/DCM was removed under a stream of nitrogen to yield the ammonium trifluoroacetate salt of the polymer. All polymer Boc deprotections proceeded in near‐quantitative yield as determined by monitoring the disappearance of the Boc ^1^H NMR signal at 1.45 ppm in CDCl_3_.

### Copolymer Post‐Functionalization with Squaramides (**PSq1 – PSq4**)

Copolymers (30 – 100 mg) (**P3** for **PSq1** and **PSq3**, **P4** for **PSq2** and **PSq4**) were dissolved in MeOH:DMF (10 mL, 1:1 *v/v*) and Et_3_N (0.1 mL), squaramate (**Sqt1** or **Sqt2**, 2 equiv. relative to AEMA monomer) and Zn(OTf)_2_ (0.2 mol equiv.) was added. The solution was heated to 90 °C and stirred for 12 h. The solution was allowed to cool and the solvent was removed under a stream of N_2_ to give a yellow residue, which was purified via dialysis in DMF:acetone (1:1 *v/v*) to yield clean copolymers **PSq1 – PSq4** (yield of squaramide conversion 90%).

## Conflict of Interest

The authors declare no conflict of interest.

## Supporting information

Supporting Information

## Data Availability

The data that support the findings of this study are available from the corresponding author upon reasonable request.
