# Supplementary material for: Water‐Soluble Squaramide‐Functionalized Copolymers for Anion Recognition
Source: Macromol Rapid Commun. 2023 Oct 2;46(8):2300406. doi: 10.1002/marc.202300406 (PMC12004901; doi:10.1002/marc.202300406)
Supplement: Supplementary file 1 — Supporting Information [file MARC-46-2300406-s001.pdf]

Macromolecular Rapid Communications

Supporting Information

for *Macromol. Rapid Commun.*, DOI 10.1002/marc.202300406

Water-Soluble Squaramide-Functionalized Copolymers for Anion Recognition

Jakob D. E. Lane, Gabrielle Shiels, Parathan Ramamurthi, Markus Müllner and Katrina A. Jolliffe**

Supporting Information

Water-soluble squaramide-functionalised copolymers for anion recognition

Jakob D. E. Lane, Gabrielle Shiels, Parathan Ramamurthi, Markus Müllner and Katrina A. Jolliffe**

Contents

Materials 3
Methods 3
Supplementary experimental section (small molecules) 5
Supplementary schemes and table 8
NMR spectra of copolymers 10
Anion titrations 12
References 15

Materials

All chemicals and solvents were of reagent grade (>95%) and used as received unless otherwise noted.

Methods

Reactions were magnetically stirred and were monitored by thin-layer chromatography (TLC) carried out on Merck alumina silica gel plates (60F-254) using UV light as visualising agent.

Nuclear magnetic resonance (NMR) spectra were recorded at 300 K using either a 600 Bruker Avance (equipped with a high resolution cryogenic triple nucleus probehead), 500 Bruker Avance DPX 400 or a Bruker Avance 300 spectrometer. NMR spectra were calibrated to the residual proton solvent peak in $CDCl_3$ (δ 7.26 ppm) or DMSO-d_6 (δ = 2.50 ppm) at 300 K. ^{1}H NMR and ^{13}C NMR spectra were recorded at the indicated frequencies. Chemical shifts are expressed as parts per million (ppm) and are referenced to solvent residual signals. The data are reported as chemical shift, multiplicity (br = broad, s = singlet, d = doublet, t = triplet, m = multiplet), coupling constant *J* in Hz and relative integral.

Melting points (mp) were observed manually using a Stanford Research Systems Optimelt apparatus.

Mass spectrometry. Low resolution mass spectra were recorded on a Bruker amaZon SL mass spectrometer using electrospray ionisation (ESI, positive or negative mode). High resolution mass spectra were recorded on a Bruker Apex II Fourier Transform Ion Cyclotron Resonance (FTICR) mass spectrometer with a 7.0 T magnet, fitted with an off-axis Analytic electrospray source with quadrupole mass analyser, and are reported as *m/z* (relative intensity).

Infrared (IR) absorption spectra were recorded on a Bruker Alpha-E FT-IR spectrometer using attenuated total reflection (ATR) of either a solid or a thin film. Notable vibrational wavenumbers are recorded in cm^{-1}.

UV-Vis spectra were recorded on a Cary 400 UV-Vis spectrophotometer at 25 °C in a 1 cm quartz cuvette after background subtraction of the cuvette and solvent.

Fluorescence spectra were recorded on a Horiba Duetta fluorescence and absorbance spectrophotometer with temperature controlled enabled (25 °C) in a 1 cm quartz cuvette.

Size exclusion chromatography (SEC) was performed using a Shimadzu Prominence Chromatograph set at a flow rate of 1.0 ml/min at 50 °C calibrated to a polymethylmethacrylate (PMMA) standard. Samples were prepared by dissolution in DMAc (Dimethylacetamide)

containing LiBr (0.03 % w/w) and butylated hydroxytoluene (0.05 % *w/w*) and passing through a 0.22 μm syringe filter before injection.

NMR titrations were performed by additions of aliquots of the putative anionic guest as the tetrabutylammonium (TBA) salt (0.1 – 0.2 M) made up in a solution of the receptor (2.5 – 4.0 mM) in DMSO-d_6 (1% water). ^{1}H NMR spectra were recorded on a Bruker Avance DPX 500 spectrometer or a Bruker Avance DPX 400 spectrometer and calibrated to the residual proton solvent peak in DMSO-d_6 (δ = 2.50 ppm) at 300 K. Stack plots were made using MestReNova Version 6.0. Where possible, non-linear least-square curve fitting of the titration data to a 1:1 binding model using Bindfit v0.5 enabled the calculation of association constants (K_a).[1, 2] The obtained K_a values represent the average of two independent titrations. The fitting error from Bindfit is shown for each K_a value, and experimental error is estimated at less than 15% for each K_a value obtained.

UV-Vis anion binding titrations were performed by additions of aliquots of the putative anionic guest as the TBA salt (10 – 30 mM) made up in a solution of the receptor (20 – 25 μM) in DMSO (1% water). UV-Vis spectra were recorded on a Cary 400 UV-Vis spectrophotometer at 25 °C in a 1 cm quartz cuvette after background subtraction of the cuvette and solvent. The solution was stirred after each addition. Binding affinities were obtained from a global fit of the absorbance data between 330 – 430 nm, fitting to a 1:1 binding model in Bindfit. The obtained K_a values represent the average of two independent titrations, and experimental error is estimated to be less than 15% for each K_a value obtained.

Fluorescence anion binding titrations were performed by additions of aliquots of the putative anionic guest as the TBA salt (10 – 30 mM) made up in a solution of the receptor (20 – 25 μM) in DMSO (1% water). HCO_3^- was used as the TEA salt. Fluorescence spectra were recorded on a Horiba Duetta fluorescence and absorbance spectrophotometer with temperature controlled enabled (25 °C) in a 1 cm quartz cuvette. The solution was stirred after each addition. Binding affinities were obtained from a global fit of the absorbance data between 330 – 430 nm, fitting to a 1:1 binding model in Bindfit. The obtained K_a values represent the average of two independent titrations, and experimental error is estimated to be less than 15% for each K_a value obtained.

Supplementary experimental section (small molecules)

3-((3,5-bis(trifluoromethyl)phenyl)amino)-4-ethoxycyclobut-3-ene-1,2-dione **(Sqt1)**

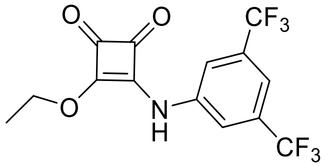

3,5-(Bis)trifluoromethylaniline (200 mg, 1.2 mmol) was added dropwise to a solution of 3,4-dimethoxy-3-cyclobutene-1,2-dione (245 mg, 1.1 mmol) in EtOH (15 mL) and the mixture was stirred at room temperature for 12 hours. The yellow precipitate that formed was collected by vacuum filtration, to give 3-((3,5-bis(trifluoromethyl)phenyl)amino)-4-ethoxycyclobut-3-ene-1,2-dione (266 mg, 70%). Mp: 207–209 °C; ^{1}H NMR (400 MHz, $CDCl_3$): 11.20 (s, 1H), 8.04 (s, 2H), 7.09 (s, 1H), 4.81 (q, *J* = 7.1, 2H), 1.43 (t, J= 7.1, 3H); ^{13}C NMR (100 MHz, DMSO-*d6*) δ 187.44, 184.50, 179.26, 169.17, 140.20, 131.38 (q, J = 59 Hz,) 128.51, 124.89, 119.39, 117.66, 116.22, 70.13, 15.33; IR (solid): ν_{max} = 3250, 3100, 1814, 1715, 1560, 1374, 1277, 1128 cm^{-1}; MS (ESI) *m/z*: 354 [M+H]$^+$. The spectral data were in good agreement with those reported in the literature.[3, 4]

3-((9H-Fluoren-2-yl)amino)-4-ethoxycyclobut-3-ene-1,2-dione **(Sqt2)**

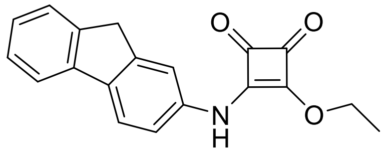

A mixture of 3,4-dimethoxy-3-cyclobutene-1,2-dione (50 mg, 0.30 mmol) and zinc trifluoromethanesulfonate (12 mg, 0.03 mmol) was stirred in methanol (10 mL). 2-Aminofluorene (52 mg, 0.28 mmol) was added and the mixture was stirred at room temperature. After 48 hours, the precipitate was collected by filtration and the solid was washed with cold methanol to give **119** as a yellow solid (55 mg, 65%). Mp: 212–215 °C; ^{1}H NMR (400 MHz, DMSO-d_6): δ 10.80 (s, 1H), 7.86-7.83 (t, *J* = 5 Hz, 2H), 7.60 (s, 1H), 7.58–7.56 (d, *J* = 10 Hz, 1H) 7.41–7.36 (m, 2H), 7.31–7.27 (td, *J* = 5, 2, 1H), 4.82–4.78 (q, *J* = 5 Hz, 2 H), 3.92 (s, 2H), 1.46– 1.43 (t, *J* = 5 Hz, 3H); ^{13}C NMR (125 MHz, DMSO-d_6): δ 188.5, 184.1, 178.7, 170.0, 144.5, 143.3, 141.1, 137.8, 137.4, 127.3, 126.9, 125.5, 120.8, 120.1, 119.2, 117.1; IR (solid) ν_{max}: 3238, 3196, 3112, 3022, 2979, 2934, 1792, 1704, 1603, 1574, 1514, 1469, 1436, 1377, 1347, 1311, 1212, 1180, 1071. 810, 765, 613 cm^{-1}; HRMS (ESI) *m/z*: calc'd for $C_{19}H_{16}O_2N_2$ [M+H]$^+$ 306.1122; found 306.1125.

3-((3,5-Bis(trifluoromethyl)phenyl)amino)-4-(butylamino)cyclobut-3-ene-1,2-dione **(Sq1)**

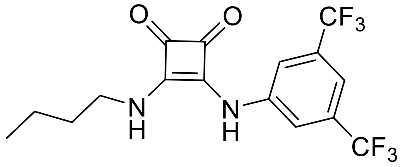

To a suspension of 3-((3,5-bis(trifluoromethyl)phenyl)amino)-4-ethoxycyclobut-3-ene-1,2-dione (100 mg, 0.28 mmol) in MeOH (10 mL), n-butylamine (25 mg, 0.34) was added. The mixture was stirred for 16 hours and the precipitate filtered and washed with cold MeOH to give a white solid (89 mg, 83%). M.p. 258 – 260; ^{1}H NMR (400 MHz, DMSO-d_6): δ 10.15 (s, 1H), 8.02 (s, 1H), 7.71 (s, 1H), 7.66 (s, 1H), 3.63 (br, 2H), 1.60–1.53 (m, 2H), 1.39–1.34 (m, 2H), 0.94–0.90 (t, J = 8 Hz); ^{13}C NMR (100 MHz, DMSO) δ 184.6, 180.2, 169.7, 162.2, 141.1, 131.1, 123.2, 117.9, 114.5, 32.5, 18.9, 13.3; MS (ESI) *m/z:* 379 $[M-H]^+$. MS (ESI): *m/z* 403 $[M+Na]^+$. The spectral data were in good agreement with those previously reported.[5]

3-((9H-Fluoren-2-yl)amino)-4-(butylamino)cyclobut-3-ene-1,2-dione **(Sq2)**

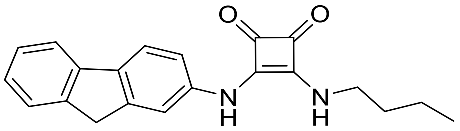

3-((9H-Fluoren-2-yl)amino)-4-ethoxycyclobut-3-ene-1,2-dione (20 mg, 0.07 mmol), butylamine (7 mg, 0.1 mmol) zinc trifluoromethanesulfonate (4 mg, 0.01 mmol) were stirred in methanol (10 mL) and the solution heated to reflux for 24 hours. The solution was allowed to cool to room temperature and the yellow precipitate was collected by vacuum filtration, and washed with cold methanol to afford **113** as a yellow solid (18 mg, 83%). Mp: 319 °C (decomposition); ^{1}H NMR (400 MHz, DMSO-d_6): δ 9.64 (s, 1H), 7.84–7.81 (m, 2H), 7.69 (s 1H), 7.59 (s, 1H), 7.59–7.55 (d, J = 10.1 Hz, 1H), 7.42–7.40 (dd, J = 10.1 Hz, 1H), 7.38–7.35 (t, J = 5.2 Hz, 1H), 7.28–7.25 (td, J = 5.2 Hz, 1H), 3.92 (s, 2H), 3.66–3.62 (q, J = 6.8 Hz, 2H), 1.62–1.56 (m, 2H), 1.43–1.36 (m, 2H), 0.96–0.93 (t, 8.0 Hz, 3H); ^{13}C NMR (100 MHz, DMSO-d_6): δ 184.5, 180.9, 169.8, 163.9, 145.0, 143.1, 141.3, 138.6, 136.4, 127.2, 126.5, 125.4, 121.1, 120.0, 117.6, 115.5, 43.9, 37.0, 33.1, 19.5, 13.9; IR (solid) ν_{max}: 3178, 3116, 3040, 2956, 2932, 2871, 1792, 1656, 1601, 1560, 1431, 1353, 1130 1095, 951, 871, 781, 764, 729, 729 cm^{-1}; HRMS (ESI) *m/z*: calc'd for $C_{21}H_{21}N_2O_2$ $[M+H]^+$ 333.1598, found 333.1597.

tert-Butyl (2-hydroxyethyl)carbamate **(Boc-EA)**

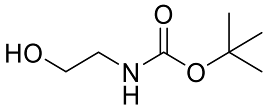

A solution of di-*tert*-butyl dicarbonate (900 mg, 4.1 mmol) in dichloromethane (5 mL) was slowly added to a solution of ethanolamine (230 mg, 3.8 mmol) in dichloromethane (20 mL) and the solution stirred for 6 hours at 25 °C. The resultant mixture was washed with saturated aqueous $NaHCO_3$ solution (3 x 20 mL), dried with $MgSO_4$, filtered and the solvent subsequently removed *in vacuo* to obtain *tert*-butyl (2-hydroxyethyl)carbamate as a colourless oil (520 mg, 86%). ^{1}H NMR (400 MHz, $CDCl_3$): δ 3.70 ppm (t, J = 1.3 Hz, 2H), 3.29 (t, J = 1.3 Hz, 2H), 1.45 (s, 9H); %). ^{13}C NMR (100 MHz, $CDCl_3$): 156.8, 79.6, 62.1, 43.0, 28.3; MS (ESI) *m/z*: 184 $[M+Na]^+$. The spectral data were in good agreement with that reported by Pasquino *et al.*[6]

2-((*tert*-Butoxycarbonyl)amino)ethyl methacrylate **(Boc-AEMA)**

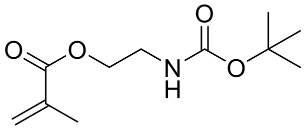

Methacryloylchloride (195 mg, 1.9 mmol) was added to a solution of **Boc-EA** (200 mg, 1.2 mmol) and triethylamine (0.3 mL, 1.9 mmol) dichloromethane (10 mL) at 0°C. The mixture was allowed to warm to 25 °C with stirring, and stirred for a further 12 hours. The pink solution was sequentially washed with water (20 mL), 10% aqueous K_2CO_3 solution (20 mL), saturated $NaHCO_3$ (20 mL), and brine (20 mL). The organic layer was dried over $MgSO_4$, evaporated under reduced pressure, and crystallized from dichloromethane/hexane (1:1, *v/v*) to give pale pink crystals of 2-(*tert*-butoxycarbonylamino)ethyl methacrylate (155 mg, 55%). Mp: 81–83 °C; ^{1}H NMR (400 MHz, $CDCl_3$): δ 6.12 (s, 1H), 5.59 (s, 1H), 4.76 (s, 1H), 4.21 (t, J = 5.2 Hz, 2H), 3.45 (m, 2H), 1.95 (s, 3H), 1.45 (s, 9H); ^{13}C NMR (100 MHz, $CDCl_3$): δ 167.1, 155.7, 135.9, 125.7 79.3, 63.7, 39.5, 28.3, 18.1; IR (solid): ν_{max} = 3384, 2979, 1690, 1633, 1523, 1248, 1160, 601 cm^{-1}; MS (ESI) *m/z*: 252 $[M+Na]^+$. The spectral data were in good agreement with that reported by Pasquino *et al.*[6]

Supplementary schemes and table

Scheme **S1**: Synthesis of squaramates Sqt1 – Sqt 3.

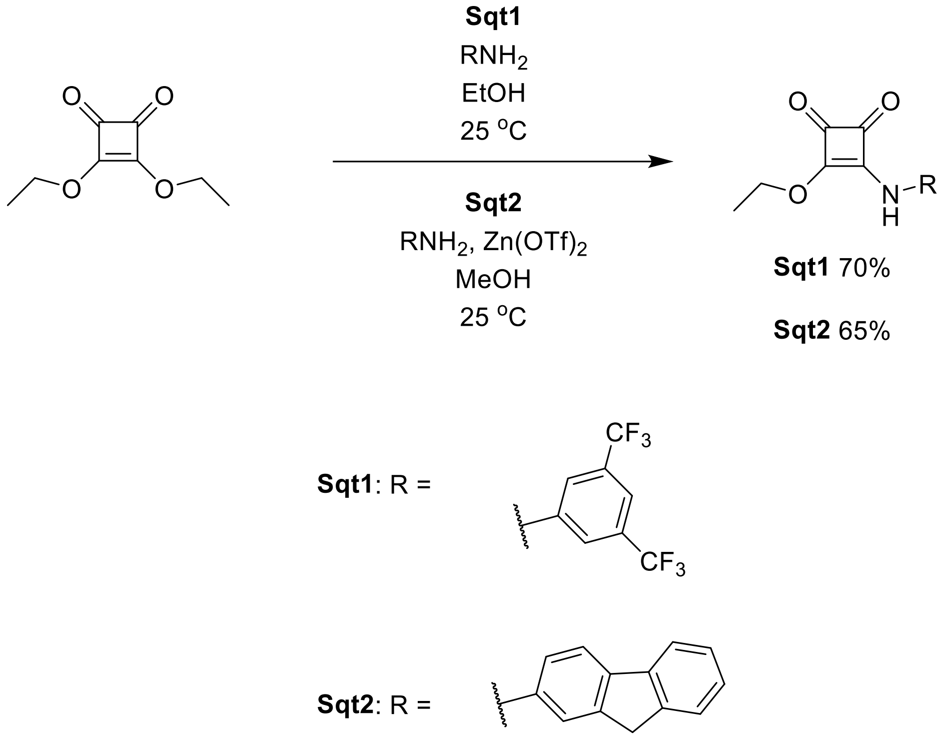

Scheme **S2**: Synthesis of squaramides **Sq1** and **Sq2**.

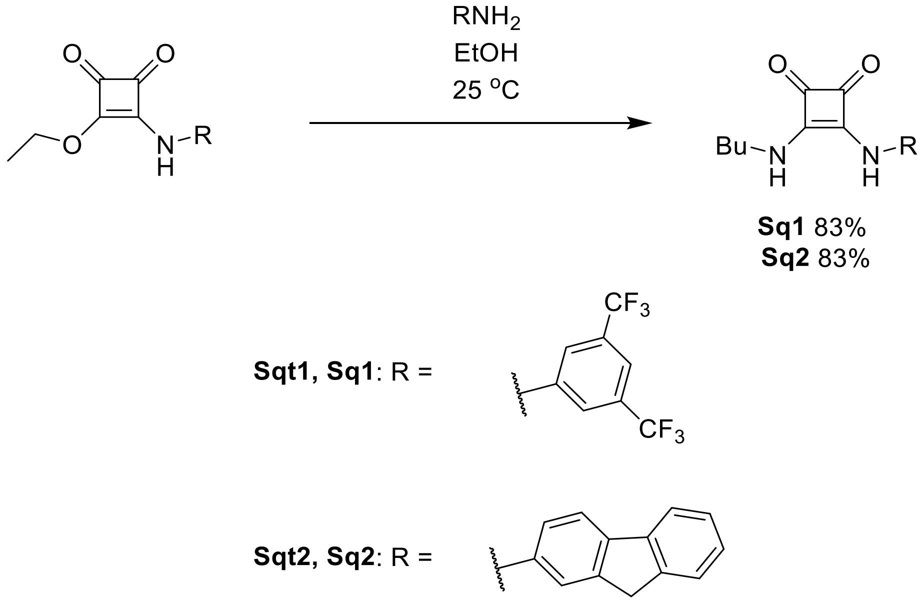

Scheme **S3**: Synthesis of Boc-EA and Boc-AEMA

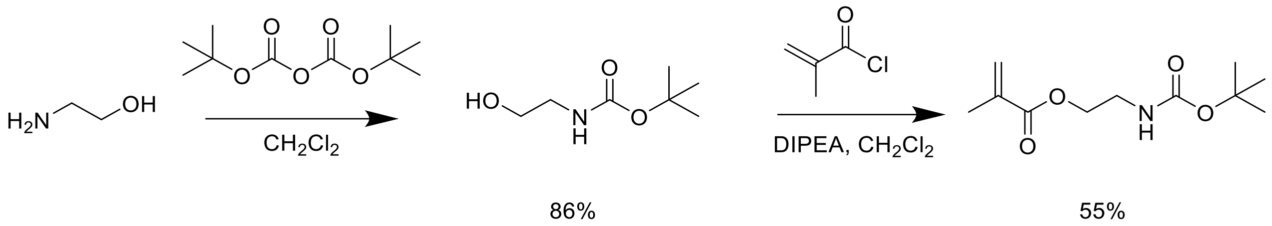

Scheme **S4**: Synthesis of P1 via ATRP polymerisation

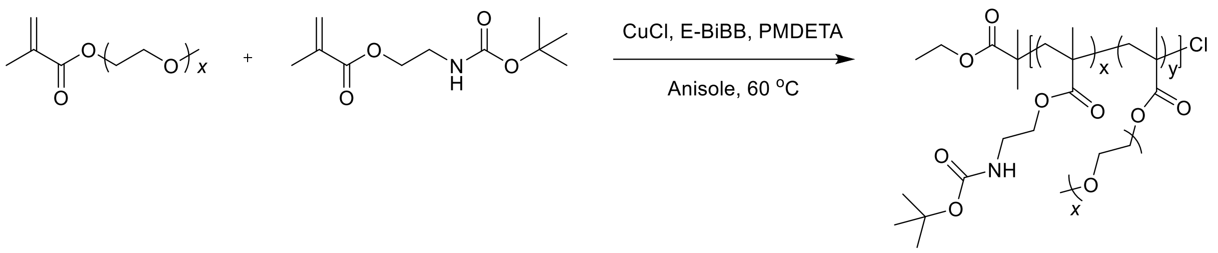

Table **S1**: Optimisation of reaction conditions for squaramide formation on polymer. Reaction time 12 h.

**Entry**	**Solvent**	**Additive**	**Temperature (°C)**	**Yield (%)**
**1**	MeOH	-	25	50
**2**	MeOH	$Zn(OTf)_2$ (20%)	65	70
**3**	MeOH/DMF (1:1, *v/v*)	$Zn(OTf)_2$ (20%)	90	>90

NMR spectra of copolymers

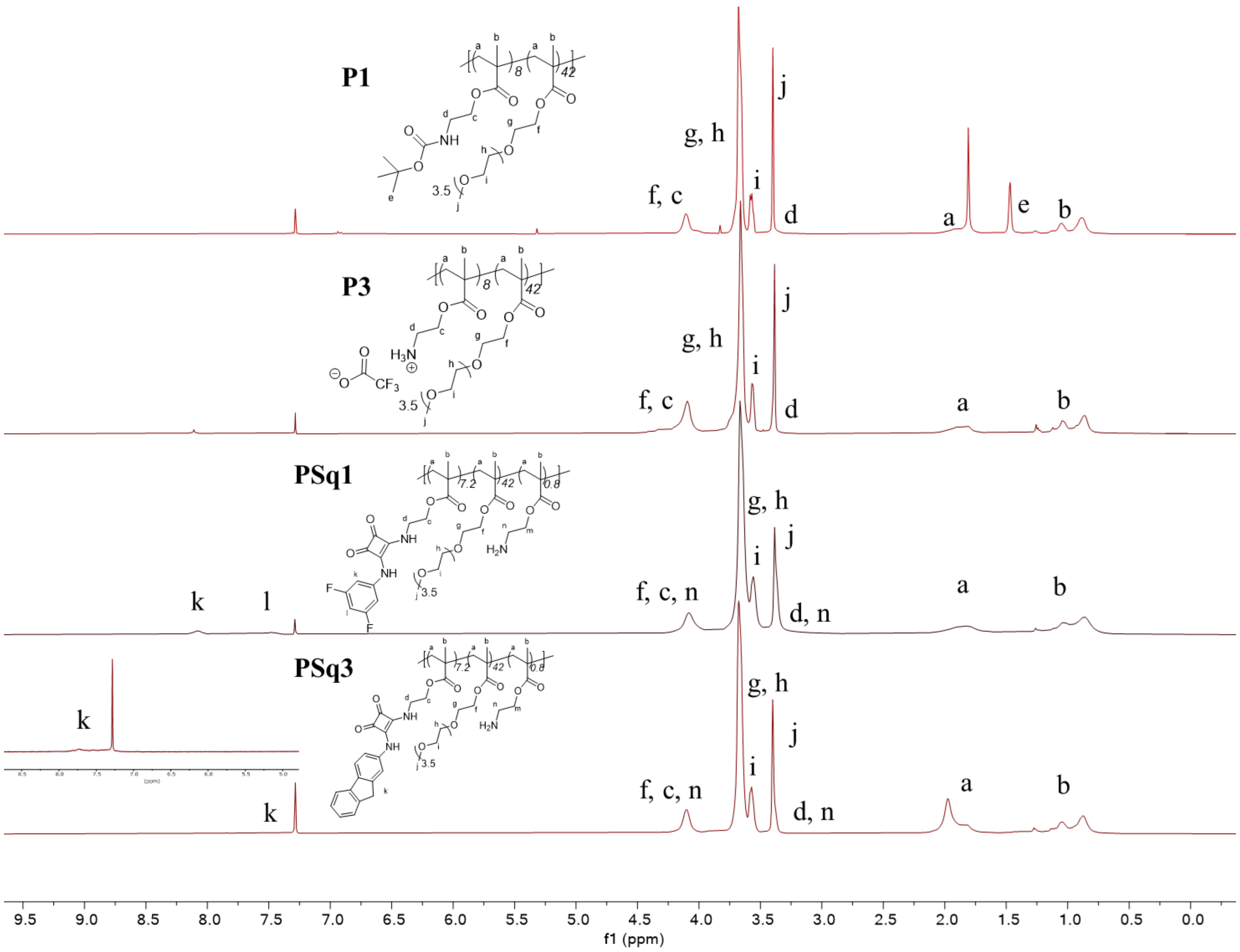

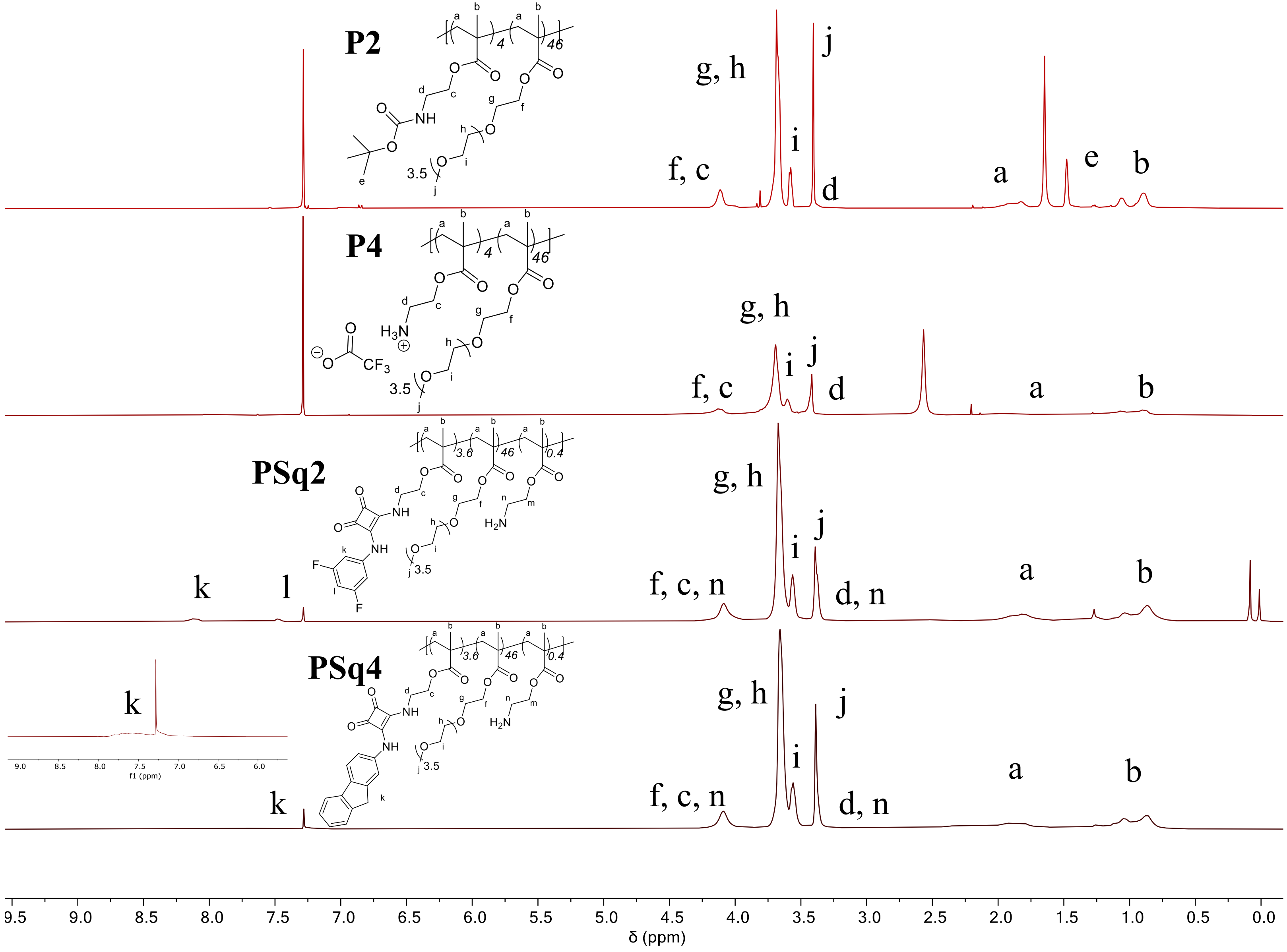
P2
g, h
j
i
f, c
d
a
e
b
P4
g, h
j
i
f, c
d
a
b
PSq2
g, h
j
i
k
l
f, c, n
d, n
a
b
PSq4
k
g, h
j
i
k
f, c, n
d, n
a
b
δ (ppm)

Anion titrations

Table **S2**: Association constants (K_a, M^{-1}) of **Sq1** and **Sq2** for anions determined by UV-Vis spectroscopic titration in DMSO (1% water). [a]Weak binding, no association constant determined. [b]Binding data could not be fit successfully to a simple binding model. Binding data for Sq taken from reference.[7]

Anion	Sq2	Sq3
**Cl^-**	-[a]	400[7]
**$H_2PO_4^-$**	4800	3900[7]
**AcO^-**	-[b]	5200[7]
**SO_4^{2-}**	-[b]	-[b, 7]

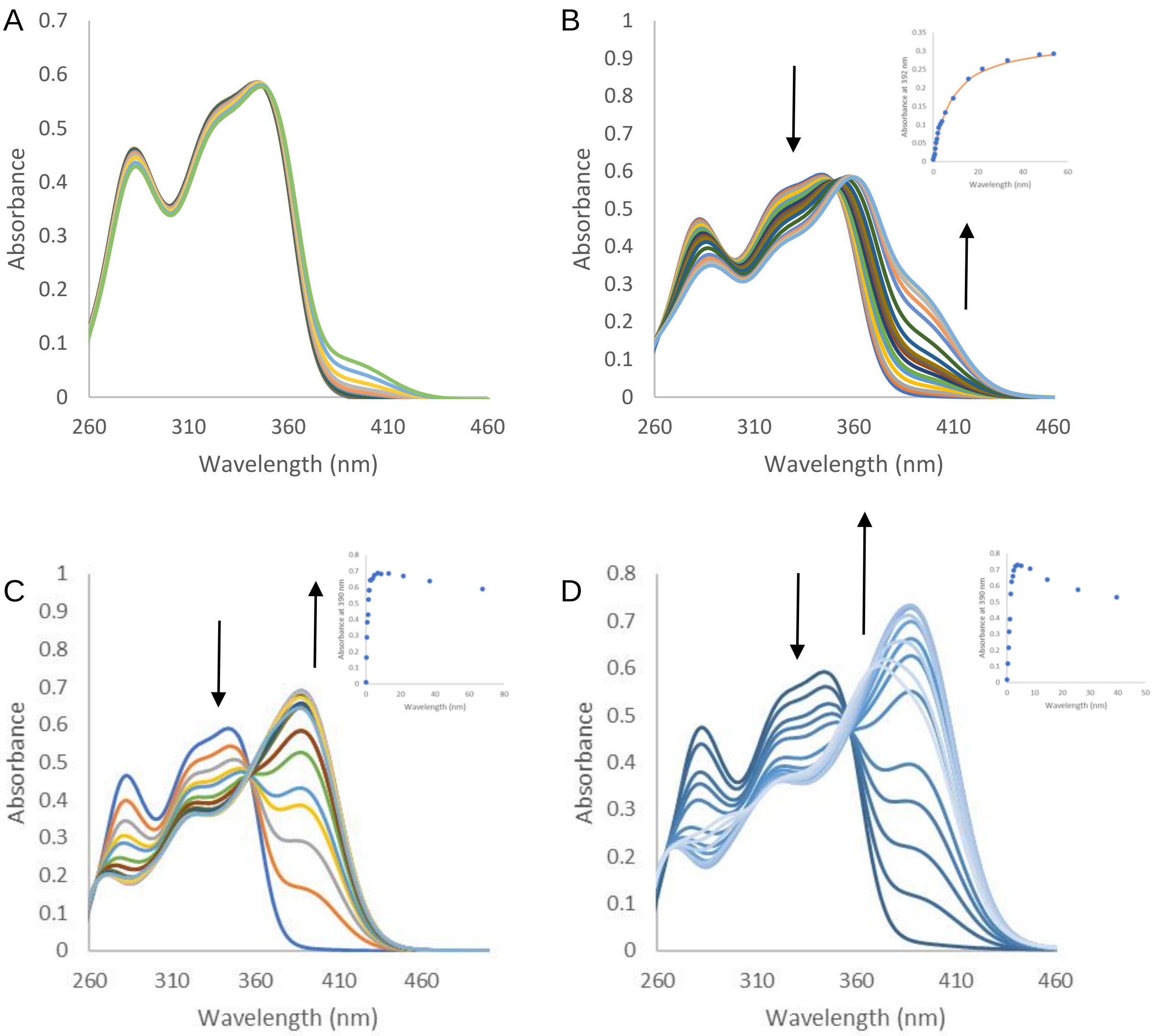

Figure **S1.** UV-Vis spectra recorded over the course of a titration of Sq1 (20 uM) with (A) TBACl, (B) $TBAH_2PO_4$, (C) TBAOAc, (D) TBA_2SO_4 (0 – 65 equivalents) in DMSO (1% water) at 298 K. Inserts show change in absorbance at maximum absorbance and 1:1 binding fit for H_2PO_4.

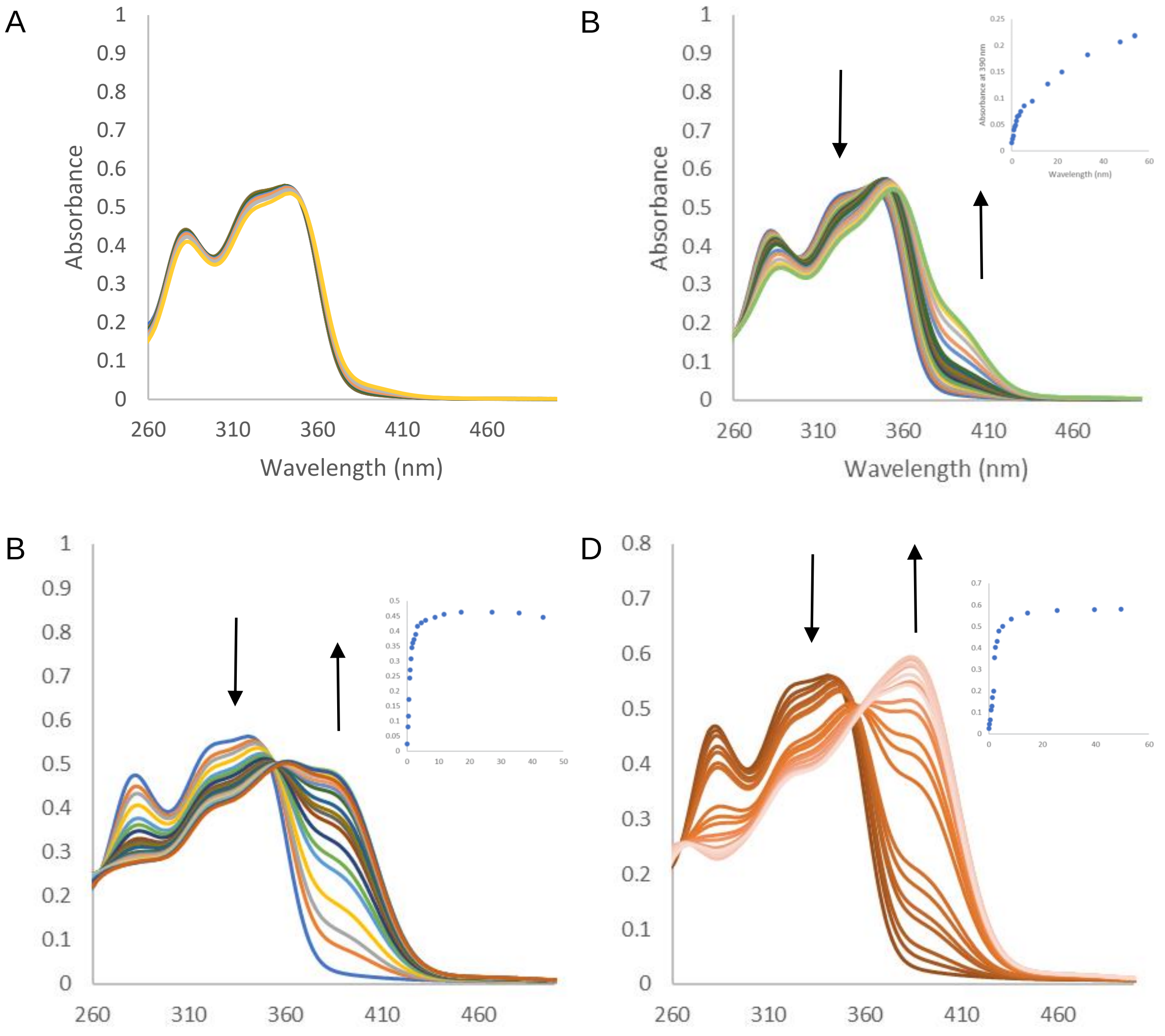

Figure **S2**. UV-Vis spectra recorded over the course of a titration of SqP1 (20 uM of squaramide) with with (A) TBACl, (B) $TBAH_2PO_4$, (C) TBAOAc, (D) TBA_2SO_4 (0 – 52 equivalents) in DMSO (1% water) at 298 K. Inserts show change in absorbance at maximum absorbance.

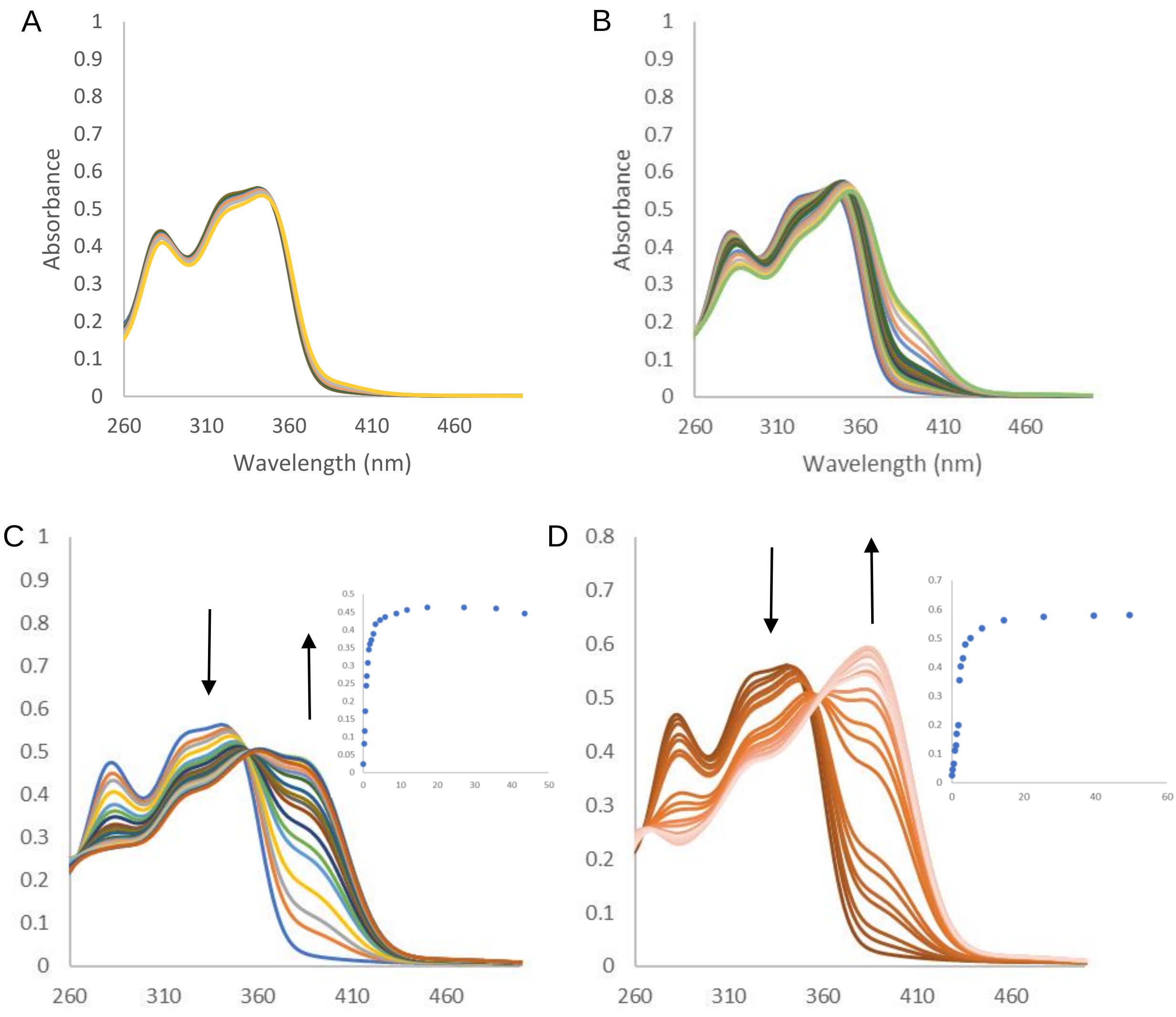

Figure S3: UV-Vis spectra recorded over the course of a titration of PSq1 (20 µM of squaramide) with (A) TBACl, (B) $TBAH_2PO_4$, (C) TBAOAc, (D) TBA_2SO_4 (0 – 1.0 mM of anion, 0 – 52 equivalents) in DMSO (1% water) at 298 K. Inserts show change in absorbance at maximum absorbance.

References

1. Thordarson, P., *Chem. Soc. Rev.* **2011,** *40* (3), 1305-1323.
2. Brynn Hibbert, D.; Thordarson, P., *Chem. Commun.* **2016,** *52* (87), 12792-12805.
3. Bao, X.; Wu, X.; Berry, S. N.; Howe, E. N. W.; Chang, Y.-T.; Gale, P. A., *Chem. Commun.* **2018,** *54* (11), 1363-1366.
4. Rostami, A.; Colin, A.; Li, X. Y.; Chudzinski, M. G.; Lough, A. J.; Taylor, M. S., *J. Org. Chem.* **2010,** *75* (12), 3983-3992.
5. Sopeña, S.; Martin, E.; Escudero-Adán, E. C.; Kleij, A. W., *ACS Catalysis* **2017,** *7* (5), 3532-3539.
6. Pasquino, R.; Zhang, B.; Sigel, R.; Yu, H.; Ottiger, M.; Bertran, O.; Aleman, C.; Schlüter, A. D.; Vlassopoulos, D., *Macromolecules* **2012,** *45* (21), 8813-8823.
7. Lane, J. D. E.; Jolliffe, K. A., *Organic & Biomolecular Chemistry* **2023**.
